# Effects of infection prevention and control measures on patient and visitor violence against health workers in China during COVID-19 pandemic

**DOI:** 10.3389/fpubh.2023.1140561

**Published:** 2023-06-05

**Authors:** Ke Su, Cheng Zhang, Ju Huang

**Affiliations:** ^1^Xuzhou Infectious Diseases Hospital, Xuzhou, Jiangsu, China; ^2^Institute of Medical Information, Chinese Academy of Medical Sciences and Peking Union Medical College, Beijing, China

**Keywords:** infection prevention and control measures, COVID-19, health workers, patient and visitor violence, workplace violence, DID

## Abstract

**Objective:**

To examine trends in patient and visitor violence (PVV) among large public hospitals from 2016 to 2020 in China, and investigate the effects of infection prevention and control (IPC) measures on PVV during the COVID-19 pandemic.

**Methods:**

The hospital-level data of PVV used in this study from 2016 to 2020 in three cities in northern China were extracted from the database of the Medical Quality and Safety Notification System from 41 public hospitals. The difference-in-difference (DID) method was used to estimate the effects of IPC measures on PVV. The empirical strategy was to compare changes in the incidence rate of PVV in public hospitals where IPC measures were stricter to relatively weaker hospitals.

**Results:**

From 2019 to 2020, the incidence rate of PVV decreases from 4.59 to 2.15% for high-IPC measure level hospitals and increases from 4.42 to 4.56% for medium-IPC measure level hospitals. The results from the DID models showed that as the IPC measure level increased, the incidence rate of PVV (*β* = −3.12, 95% CI = −5.74 ~ −0.50) decreased more significantly based on controlling for hospital fixed effects and time trends.

**Conclusion:**

The multi-dimensional and comprehensive IPC measures throughout the pandemic in China have not only controlled the pandemic, but also directly or indirectly reduced the incidence rate of PVV by alleviating the stress of health workers and the crowded working environment, creating a good order of admission, and reducing patient waiting time.

## Instruction

Incidents of violence and harassment against health workers (HWs) have been increasing during the COVID-19 pandemic (1, 2). Evidence shows that a number of occupational risks were exacerbated by the COVID-19 pandemic (3). Due to highly stressful and overcrowded work environments, heavy workloads, limited communication among multidisciplinary team members, inadequate knowledge of the epidemic, and a lack of personal protective equipment (PPE) and guidelines on the diagnosis and treatment for patients in the early stage of the COVID-19 pandemic, clinicians were exposed to an elevated risk of infection, burnout, mental health problems, and even workplace violence (WPV) (4, 5). Comprehensive studies in the Americas, Asia, and Egypt show that almost half (47%) [95% CI: (34, 61)] of HWs experienced at least one manifestation of WPV during COVID-19 (6). HWs in the USA reported a 49.4% prevalence of WPV in a 5-month period during the COVID-19 pandemic in 2020 (7). Brazil nurses reported a 51.1% prevalence (8-month period) in 2020 (8). In Egypt, the 6-month incidence of physical WPV was 9.6% and psychological WPV was 42.6% among HWs in 2020 (9). Some recent investigation studies have estimated the 2-month prevalence of WPV among HWs during the COVID-19 pandemic in China to be between 17.9 and 19.3% (10). Chinese emergency department clinicians reported a 29.2% [95% CI: (26.5, 31.9)] prevalence (1-month period) in 2020 (5). Violence is identified as one of the occupational risks amplified by COVID-19 among HWs. Numerous studies have shown that the main perpetrators of WPV in hospitals are the patients and visitors (11). We should pay more attention to the occupational health of HWs during the COVID-19 pandemic, risk assessment, and introduction of appropriate measures, especially for protection against patient and visitor violence (PVV).

The World Health Organization (WHO) and International Labour Organization (ILO) issued the guideline *COVID-19: Occupational health and safety for HWs* in February 2021, which introduced the primary prevention of COVID-19 among HWs based on risk assessments and the introduction of appropriate measures (Table 1). According to the guideline, workplace risk levels are classified as lower, medium, high, and very high risk, and infection prevention and control (IPC) measures are recommended for the different risk levels. In China, a joint prevention and control mechanism was also launched (12) and the most comprehensive and rigorous prevention and control strategy against the pandemic was enforced in areas of the COVID-19 pandemic for HWs, which was based on the *Occupational Safety and Health in Public Health Emergencies: A Manual for Protecting Health Workers and Emergency Responders published by the ILO and WHO in formulating its decisions*. Based on the local epidemiological situation, the specificity of the work setting, and work tasks, different IPC measure levels were enforced in public hospitals (1). The IPC measure levels did a good job of risk communication with HWs involved in the pandemic, provided adequate PPE in sufficient quantity and quality and regular IPC training, maintained a one-meter social distance, staggered pickup, established flexible sick leave policies, and implemented engineering, environmental and administrative controls for IPC. Administrative controls are the most important components of IPC strategies, contributing to IPC by providing policies and standard operating procedures (13, 14). Although these measures effectively protect HWs from infection, the change in the treatment process and visit regulation for patients could have increased the risk of clinician-patient conflicts. Whether the IPC measures increased the incidence rate of PVV was still unknown.

**Table 1:** Workplace risk levels and job tasks for primary prevention and mitigation of occupational exposure to SARS-CoV-2 among HWs.

Risk level	Examples of job tasks	Prevention and mitigation measures
Lower risk (caution)	Administrative tasks that do not involve contact with patients and visitors or close contact with other co-workers	do personal protection and avoid gathering; observe hand and respiratory hygiene; use fabric masks
Medium risk	Jobs or tasks with close frequent contact with patients, visitors, suppliers, and co-workers but that do not require contact with people known or suspected of being infected with SARS-CoV-2	wear medical masks and other PPE according to their tasks; do not leave the area, staggered pickup; maintain a one-meter social distance
High risk	Exposure to patients with known or suspected COVID-19 and their respiratory samples; entering sites occupied by patients with known or suspected COVID-19	implement engineering, environmental and administrative controls for IPC, and provide adequate PPE in sufficient quantity and quality
Very high risk	Work with COVID-19 patients where aerosol-generating procedures are frequently performed; work with infected people in indoor, crowded places without adequate ventilation	provide regular IPC training, including on the use of PPE; establish flexible sick leave policies

PPE, personal protective equipment.

IPC, infection prevention and control.

Over the years, researchers around the world have been studying the risk factors of WPV against HWs. Previous studies have analyzed HWs and their workplace characteristics, and risk factors have been identified for gender, experiences at the present workplace, education, age, department, whether to work in a tertiary hospital, marital status, and work experience (10, 15). The regional differences observed in the prevalence of WPV may be attributed to broader social (eg., cultural attitudes to HWs, work setting, work environment, and healthcare system) and individual factors (eg., age, gender, education level, marital status, professional level, and work tenure) (16). Other studies analyzed the effect of measures on WPV and PVV. Liu et al. (17) showed that the implementation of measures can contribute to the prevention and control of WPV, and security measures were the most recognized measures (81.03%), followed by improved surroundings in second place (52.33%). The study’s findings suggest that prevention strategies are urgently needed, particularly in emergency departments, mental health, and prehospital settings, to reduce violence towards healthcare professionals in the workplace to maintain the healthcare system (16). Al-Azzam et al. (18) showed that anti-violence policies and training in dealing with violence were important predictors of WPV for mental health department nurses. However, these studies were mainly cross-sectional studies and lacked sound study design to evaluate the intervention measures and could not analyze the causal relationship between the measures and WPV or PVV (17). In addition, COVID-19 is one of the most severe global health crises that humanity has ever faced (19). Relevant studies have focused on the impact of the IPC measures on occupational infections in HWs, psychological distress, and WPV (20), but fewer studies have specifically examined PVV and its trends.

The COVID-19 pandemic has caused a large number of deaths, with a global cumulative total of 655,689,115 confirmed cases of COVID-19 pneumonia and 6,671,624 cumulative deaths as of 00:07 on January 5, 2023 (21) posing a serious threat to public health. Thus, IPC measures can be expected and the health status of HWs should be valued. In this study, the number of PVV incidents and the incidence rate of PVV during the COVID-19 pandemic in China, from 2016 to 2020 were described, and the effects of IPC measures on PVV were examined using the difference-in-difference (DID) models. The findings may inform public health policy all over the world to protect the health and safety of HWs to control the global pandemic of COVID-19 more efficiently.

## Methods

### Data sources

In total, 5 years of hospital-level data, from 2016 to 2020, from three cities, Beijing, Shijiazhuang, and Tongliao, in northern China were used in this study. The hospital-level PVV data used in this study were extracted from the database of the Medical Quality and Safety Notification System (hereafter referred to as “the Notification System”) from 41 public hospitals in these cities (22), which had been developed by the local health authority, including the number of PVV incidents and the characteristics of the hospitals and services provided. The participating hospitals are all large public hospitals.

### Sample

According to the workplace risk level table given by the WHO (Table 1), we assessed the workplace risk level of each hospital by investigating whether there were known or suspected SARS-CoV-2 infected people entering the hospital during the pandemic. During the pandemic, localities have strengthened the construction of fever, respiratory and intestinal clinics in some hospitals above the secondary level according to specific conditions, mainly including general hospitals, infectious disease hospitals (including COVID-19 designated treatment hospitals), and children’s hospitals. These hospitals were exposed to patients with known or suspected COVID-19 and their respiratory samples, therefore, HWs from these hospitals were at high risk of occupational exposure to SARS-CoV-2. Based on the definitions in Table 1, we defined the above hospitals as high risk. While for other hospitals, HWs were often in close contact with patients and visitors not exposed to SARS-CoV-2. According to the explanation in Table 1, we defined these hospitals as medium risk of occupational exposure to SARS-CoV-2. The medium-risk level hospitals include kidney hospitals, dental hospitals, psychiatric hospitals, plastic surgery hospitals, rehabilitation hospitals, and ophthalmic hospitals. In total, 23 hospitals were at the high-risk level and 18 hospitals were at the medium-risk level. The description of the characteristics of the high-risk and medium-risk hospitals is presented in Table 2. All personal identifiers (e.g., name, employer, and contact) were removed. The Notification System of the health care institutions gives a comprehensive and detailed account of PVV, which provided the required data for our study.

**Table 2:** Description of hospital characteristics.

Risk level	IPC measures level	No. of hospitals, *n* (%)		
		Type		Total
		General	Specialized	
High risk	High	19 (46.3)	4 (9.8)	23 (56.1)
Medium risk	Medium	0 (0.0)	18 (43.9)	18 (43.9)
Total		19 (46.3)	22 (53.7)	41 (100.0)

HWs, health workers.

General, general hospital.

Specialized, specialized hospital.

### Identifying the impact of IPC measures

Prior to the outbreak of COVID-19, the IPC measures were mainly for common communicable diseases, and the comprehensive COVID-19-specific IPC measures for all hospitals in China were practically nonexistent. After the outbreak of COVID-19 in 2020, different COVID-19-specific IPC measures levels were mainly implemented in different types of public hospitals, based on the local epidemiological situation, the specificity of the work setting, and work tasks. Through a document and literature review, we obtained the requirements on IPC measures for the hospitals set by the Municipal Health Commission of the cities. Broadly speaking, risk levels and IPC measure levels were determined based on the likelihood of HWs being exposed to known or suspected COVID-19 patients. The hospitals with a high IPC measures level were able to treat COVID-19 patients with high workplace risk levels and implemented the strictest engineering, environmental and administrative controls for IPC. The hospitals with a medium IPC measures level had a medium workplace risk and introduced measures to wear medical masks and other PPE according to their tasks and maintained a one-meter social distance.

### Statistical analysis

In this study, the generalized DID method was used to evaluate the effects of IPC measures on PVV during the epidemic. A DID model is mainly used in research to estimate the causal effect of an intervention by comparing changes over time in an outcome variable between a treatment group and a control group, and it is a simple and well-developed approach that is gradually being used in a wide range of fields (23). The empirical strategy is to compare changes in PVV incidence in hospitals where IPC measures were stricter to institutions that had weaker measures. The difference between our estimates and a standard DID strategy is that we use continuous measures of the intensity of treatment and thereby capture more variation in the data (24). Since this approach does not require capturing any effect of IPC measures on blank control groups compared to the traditional DID method, it will underestimate the full impact of anti-pandemic measures less. Of course, different IPC measure levels are not randomly assigned. Documents of prevention and control strategy indicate that type and scale status can explain a substantial share of this variation. Therefore, the empirical approach is to look at whether there is a break in any pre-existing differences in the level or trend of PVV outcomes around the time of IPC measures being implemented in 2020. The estimating equation is

(1) $$y_{\mathrm{it}}=\alpha_{i}+\beta {\mathrm{Impact}}_{i}\times {\mathrm{year}}_{t}+\lambda_{i}+\gamma_{t}+\varepsilon_{\mathrm{it}}$$

$y_{\mathrm{it}}$ is the result variable, indicating the number or incidence of PVV in year *t* at hospital *i*. ${\mathrm{Impact}}_{i}$ is the dummy variable of the measure group, indicating the IPC measure with which the hospital was affected, ${\mathrm{year}}_{t}$ is the dummy variable of measure time, and ${\mathrm{Impact}}_{i}\times {\mathrm{year}}_{t}$ is the interaction term of the two. $\lambda_{i}$ is a series of a hospital’s individual fixed effects, $\gamma_{t}$ represents a vector of year dummies, and $\varepsilon_{\mathrm{it}}$ is the random error term. The analysis centers on two hospital-level outcomes: the number of PVV incidents and the incidence rate of PVV. These were calculated by hospital level as follows: where *i* is the *i*th institution, and *i* = 1, 2 … 41, *t* is the *t*th year, and *t =* 2016, 2017… 2020. The coefficient of interest in Equation (1) is β , which is the estimated impact of IPC measures on the incidence rate of PVV.

(2) $$\begin{array}{l} \mathrm{Incidence rate of}\;{\mathrm{PVV}}_{\mathrm{it}}=\\ \;\;\;\;\;\;\;\;\;\;\;\;\;\;\frac{\sum \mathrm{Number of}\;\mathrm{PVV}\;{\mathrm{incidents}}_{\mathrm{it}}}{\sum \mathrm{Number of}\;{\mathrm{HWs}}_{\mathrm{it}}}\times 100\% \end{array}$$

The annual total number of outpatient visits and inpatient admissions was used to estimate the HWs workload. Therefore, the indicators of outpatient workload (the average number of daily outpatient visits per doctor) and inpatient workload (the average daily inpatient admissions per doctor) were calculated. The workloads of HWs were calculated as follows: where *i* is the *i*th institution, and *i* = 1, 2,…., *N* (*N* = total sample size), *j* is the *j*th group (eg, IPC measures level and type), and *j* = 0, 1, 2… *j* (*j* = the number of institution groups). The workloads of HWs were calculated when *j* was 0 and 249 is the number of working days for the same period for doctors and 365 is the total number of days in a year (22)

(3) $${\mathrm{Outpatient workload}}_{j}=\frac{\sum \mathrm{Number of}\;{\mathrm{outpatient visits}}_{\mathrm{ij}}}{\sum \mathrm{Number}\;{\mathrm{of doctors}}_{\mathrm{ij}}\times 249}$$

(4) $${\mathrm{Intpatient workload}}_{j}=\frac{\sum \mathrm{Number of}\;{\mathrm{patient admissions}}_{\mathrm{ij}}}{\sum \mathrm{Number}\;{\mathrm{of doctors}}_{\mathrm{ij}}\times 365}$$

The associations between categorical variables were tested with chi-square tests, and *p* < 0.05 (two-tailed) was considered statistically significant.

## Results

### Distribution and prevalence of PVV

A total of 41 hospitals participated, 23 of them are in the high-risk level and 18 hospitals are in the medium-risk level. Among them, there were 19 general hospitals, and 22 specialist hospitals. The total HWs in high-risk level hospitals increased from 26,037 in 2016 to 31,996 in 2020, and the total HWs in medium-risk level hospitals increased from 8,461 in 2016 to 10,501 in 2020.

Table 3 reports the mean hospital outcomes from 2016 to 2020. Overall, the total incidence rate of PVV in the surveyed hospitals increased from 3.62% in 2016 to 4.52% in 2019 and decreased to 3.21% in 2020. Specifically, the incidence rate of PVV was higher in high-risk hospitals than in medium-risk hospitals from 2016 to 2019 and was reduced in high-risk hospitals and significantly lower than in medium-risk hospitals in 2020.

**Table 3:** Description of the hospital-level prevalence of PVV.

Hospital basic information		No. of hospitals	2016	2017	2018	2019	2020	Adjusted 2020
Violence episodes, *n*	High	23	632	706	827	1,115	566	632
	Medium	18	236	244	230	309	334	351
	Total	41	868	950	1,057	1,424	900	–
HWs, *n*	High	23	26,037	26,930	29,023	31,434	31,996	–
	Medium	18	8,461	8,848	9,466	9,776	10,501	–
	Total	41	34,498	35,778	38,489	41,210	42,497	–
PVV rate (%)	High	23	3.91	3.79	3.25	4.59	2.15	2.40
	Medium	18	3.26	2.38	2.67	4.42	4.56	4.78
	Total	41	3.62	3.17	2.99	4.52	3.21	–

PVV, patient and visitor violence; HWs, health workers.

PVV rate, Incidence rate of PVV.

High: high-IPC measures level hospitals.

Medium: medium-IPC measures level hospitals.

IPC, infection prevention and control.

Adjusted 2020, the number and incidence rate of PVV in 2020 was calculated by assuming that the HWs workload in 2020 was the same as that in 2019.

**Figure 1:**
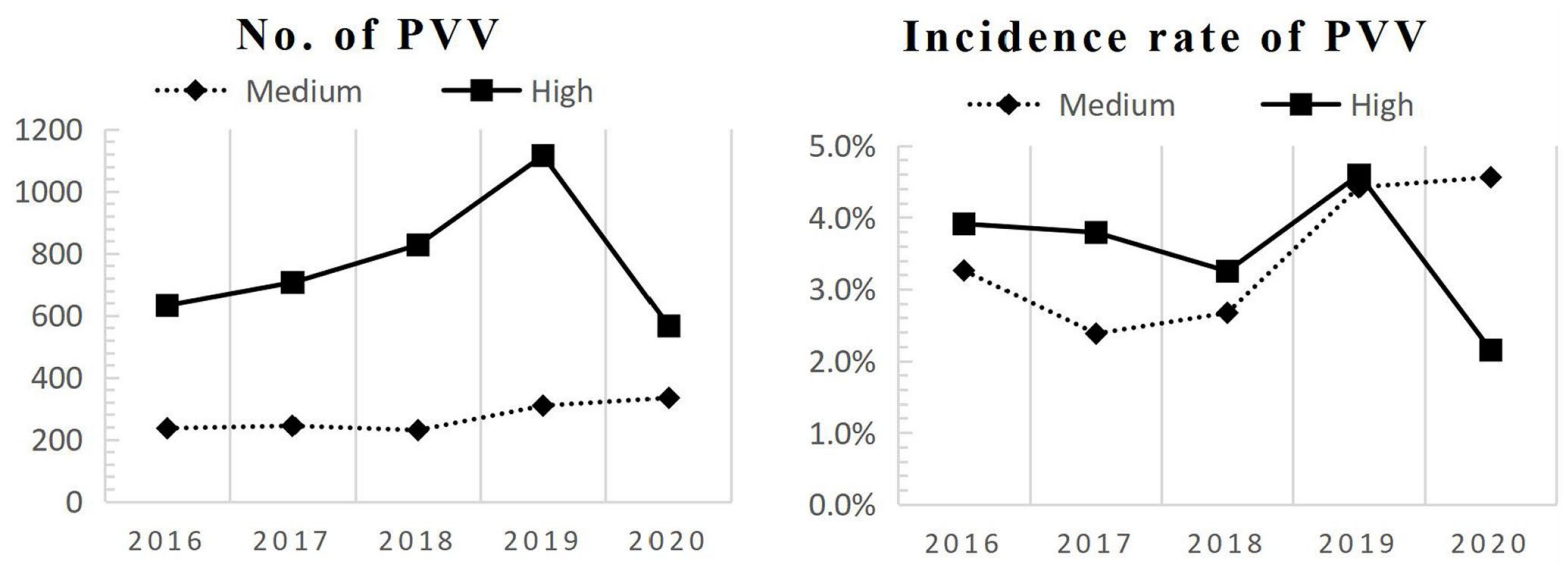
Five-year hospital time series patterns of PVV. PVV, patient and visitor violence; High, High-IPC measure level hospitals; Medium, Medium-IPC measure level hospitals; IPC, infection prevention and control.

Figure 1 shows the five-year hospital time series patterns for two PVV outcomes by IPC measures. From 2016 to 2019, the incidence rate of PVV in high-risk hospitals trended upward from 3.91 to 4.59% and declined dramatically to 2.15% in 2020. However, the incidence rate of PVV in medium-risk hospitals largely trended upward from 2016 to 2019, rising from 3.26 to 4.42%, and slightly increased to 4.56% in 2020.

### DID models of PVV

**Figure 2:**
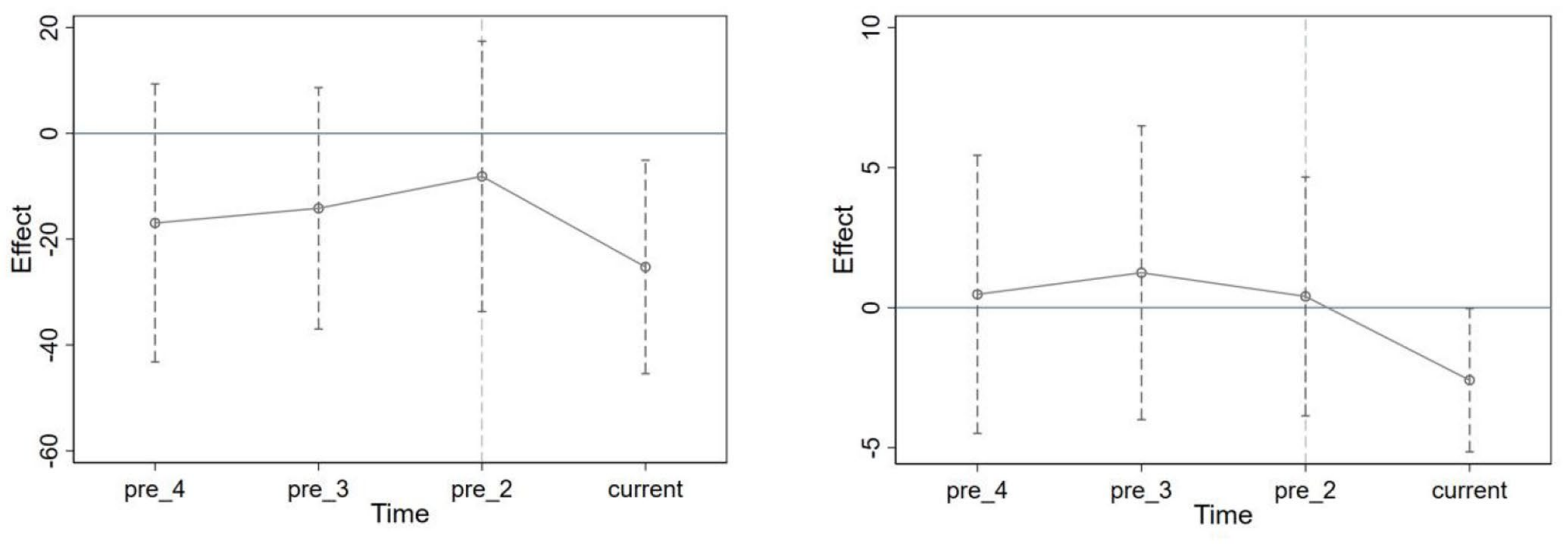
Parallel trend test of PVV. The left figure shows the parallel trend test for the number of PVV incidents, and the right figure shows the parallel trend test for the incidence rate of PVV. The 95% CI before the implementation of the measures in 2020 contains 0, which indicates that there is no significant difference between the treatment and control groups before the measures’ time point. Current = 2020. pre_2 = 2018; pre_3 = 2017; pre_4 = 2016.

To ensure that the variables had a common trend in each IPC measure level hospital before the measures were implemented, a parallel trend test was done for each of the two variables, and its results showed that there were indeed common trends before the measures were implemented (Figure 2). We find no evidence of a differential relationship between the prevalence of COVID-19 on the number of PVV incidents and the incidence rate of PVV in the pre-2020 period.

Row 1 of Table 4 shows the results of the respective DID model regression of the effects of IPC measures. We found that the DID model results for the baseline specification were that the number of PVV (*β* = −15.45, *p* = 0.006) and the incidence rate of PVV in hospitals (*β* = −3.12, *p* = 0.021) tended to decrease more significantly with the higher IPC measures level on the basis of controlling for hospital fixed effects and time trends. This means that as the hospital’s IPC measures improved, the incidence rate of PVV decreased.

**Table 4:** DID model results of PVV.

	No. of PVV			PVV rate		
	*B*	95% CI	*p*	*B*	95% CI	*p*
1. Baseline specification	−15.45	(−26.28, −4.61)	0.006	−3.12	(−5.74, −0.50)	0.021
2. Adjusted baseline specification	−13.89	(−28.86, 1.08)	0.068	−4.20	(−7.51, −0.88)	0.014
3. Beijing surveyed hospitals	−19.35	(−39.44, 0.74)	0.058	−5.23	(−10.24, −0.22)	0.042
4. Placebo test	2.77	(−4.74, 10.29)	0.126	0.77	(−1.08, 2.62)	0.414

PVV, patient and visitor violence. CI, confidence interval.

Row 1: use the valid data collected for outcome assessment.

Row 2: row 1 with adjusted data for 2020.

Row 3: row 1 without Tongliao surveyed hospitals.

Row 4: the interval is set as 2016–2018 and the year of implementation of the measure is assumed to be 2017.

### Robustness

We investigated the robustness of the preceding results (rows 2 and 3 in Table 4). Workload is positively associated with PVV (22), and a decrease in workload in Chinese public hospitals during the pandemic will affect the incidence rate of PVV, which is also associated with the IPC measure levels, thus creating a confounding effect. Therefore, we adjusted the 2020 PVV data (Table 3) based on the multivariate linear model results that the incidence rate of PVV increased by 0.236% for each unit increase in the outpatient workload of HWs, as found in a previous study (22). The number and incidence rate of PVV in 2020 was calculated after excluding the impact of workload by assuming that the HWs workload in 2020 was the same as that in 2019. The adjusted baseline specification, which is row 2 of Table 4, is the revised data obtained by adjusting the 2020 data. The incidence rate of PVV (*β* = −4.20, *p* = 0.014) of the adjusted baseline specification showed a downward trend, and the test result was statistically significant, which indicates that IPC measures still had a decreasing effect on the incidence rate of PVV in hospitals to some extent after excluding the effect of decreasing workload.

Row 3 of Table 4 shows that the result of the incidence rate of PVV was robust by excluding the Shijiazhuang and Tongliao surveyed hospitals with a low incidence rate of PVV from the sample. By analyzing the hospitals in Beijing, we found that the incidence rate of PVV (*β* = −5.23, *p* = 0.042) showed a decreasing trend, and the results were statistically significant, indicating that the IPC measures still had a certain degree of decreasing effect on the incidence rate of PVV in hospitals after excluding the effect of regional differences. Overall the results were quite robust.

However, even if the trends in the treatment and control groups were common prior to the implementation of the measures, there is still a concern about whether other policies that may have influenced the change in trend occurred at the same time, that is, the change in the trend in the treatment and control groups after the point of measures intervention may not be caused by the measures, but by other policies in the same period. Thus, row 4 shows the placebo test results. The study interval was set as 2016–2018 and the year of implementation of the measure was assumed to be 2017, and regressions were performed on the DID models (Table 4). The results showed that *β* = 0.77 (*p* > 0.05) and the difference was not statistically significant, indicating that the change in trend between the treatment and control groups after the intervention time point of the measure was indeed caused by the measures. This means there were positive effects of prevention and control measures for PVV in public hospitals during the COVID-19 pandemic.

## Discussion

To the best of our knowledge, this study is the first panel data analysis of PVV in multiple hospitals during the 2020 COVID-19 pandemic in China and PVV among HWs in the previous 4 years to examine the impact of implementing measures on the number of PVV incidents and the incidence rate of PVV. The data set was drawn from a sample of multiple hospitals and is surveillance data. The incidence rate of PVV from 2016 to 2019 was fluctuating upward and sees a rapid decline in 2020, which could be attributed to various IPC measures during the pandemic.

### The increase in the incidence rate of PVV from 2016 to 2019

The incidence rate of PVV from 2016 to 2019 was fluctuating upward, which is in line with previous studies (25). Over the years, China has made great efforts to reduce PVV in the health sector. In 2015, the Ministry of Public Security issued *Six Articles on Public Security Organs’ Maintenance of Public Order in Medical Institutions Measures*; “medical disturbance” was incorporated into the criminal law and classified as a “crime of disturbing public order” (26). The *Regulations on the Prevention and Handling of Medical Disputes* were implemented on 1 October 2018 (27), and the National Development and Reform Commission (NDRC) issued the *Memorandum of Cooperation on the Implementation of Joint Punishment for Persons Responsible for Breach of Trust that Seriously Endangers the Normal Medical Order* on 16 October 2018 (28). However, due to the uneven distribution of medical resources, most of the quality resources are concentrated in urban tertiary hospitals, and individuals who fall ill are bound to flock to tertiary hospitals in large cities (29). As a result, the workload of HWs in China’s tertiary hospitals has increased year by year. According to the local Health Statistical Yearbook from 2016–2019, the daily inpatient per doctor in tertiary hospitals in Beijing, Shijiazhuang, and Tongliao rose from 0.12 to 0.14, 0.21 to 0.22, and 0.24 to 0.30, respectively. Previous studies showed that workload is positively related to the incidence rate of PVV (22). Therefore, even though some prevention measures for PVV in health sectors were implemented, the incidence rate of PVV in hospitals shows an increasing trend from 2016–2019 as the workload continues to rise. The increased workload of HWs led to inadequate communication with patients and their visitors, more waiting time, and a lower quality of service than expected, which could have increased the risk of PVV towards HWs (25).

### The effects of IPC measures on the incidence rate of PVV

Of interest, we found that the IPC measures did not lead to an increase in the incidence rate of PVV after controlling for workload and the effects of the pandemic, despite empirical evidence that the IPC measures can lead to increased tension and violence during outbreaks (30). Accordingly, the WHO and ILO also noted that HWs may be at higher risk of PVV in the context of the COVID-19 pandemic response and that well-coordinated and comprehensive measures are needed to reduce or prevent PVV and protect the health and safety of HWs (1). These have prevented the occurrence of violence among patients and their visitors to a certain extent and safeguarded the HWs. At the same time, we found that implementing IPC measures will indeed provide more protection for HWs than not implementing IPC measures. Evidence suggests that risk factors for HWs experiencing PVV include high workload, crowded work environment, high stress, and mental health problems such as burnout and the lack of PPE (4, 5). However, IPC measures provided adequate PPE in sufficient quantity and quality and regular IPC training and established flexible sick leave policies to relieve the stress of HWs and safeguard their mental health. Administrative controls implemented in China prevented exposure to, and transmission of, infectious agents to a susceptible person, performed staggered consultation periods, limited the number of patients’ companions or visitors, alleviated crowded work environments and stress on HWs, among other things. Engineering and environmental controls increased ventilation and installed physical barriers and hand-washing facilities to prevent infection. Hospitals used electronic means to effectively relieve the work pressure of flow transfer staff, improved the efficiency of pre-screening and triage, reasonably triaged febrile patients from general patients, ensured orderly consultation, and avoided gatherings that cause cross-infection (31).

In order to provide occupational safety for HWs during the pandemic, the Chinese government has not only implemented comprehensive IPC measures, but also improved laws and regulations to protect health workers from psychological factors such as discrimination, violence, depression, anxiety, and burnout (3). For example, the Civil Code enacted in 2020 provided a clearer and more detailed delineation of the legal rights and responsibilities of healthcare providers, healthcare workers, and patients (32). The law recognizes medical institutions as public places and strengthened the public security authorities’ obligation to maintain order (33). *The Basic Medical Care and Health Promotion Law*, which came into effect in 2020, clearly prohibits any organization or individual from threatening or endangering the personal safety of medical and healthcare personnel or violating their human dignity (33). On 10 March 2020, the Supreme People’s Court released *the first batch of 10 typical cases of punishing crimes against pandemic prevention and control in accordance with the law* (34), which served as a warning to the public. The Beijing Municipal Public Security Bureau, together with the Municipal Health and Health Commission, jointly issued *the Regulations on the Management of Hospital Safety and Order* in Beijing, the results of which showed that more than 75% of medical staff believed that the phenomenon of “medical trouble” had been significantly reduced and 89% of medical staff believed that they felt more secure than before (35).

In addition, during the pandemic, more than 42,000 HWs rushed to Hubei and disregarded their personal lives (36). Medical experts played a central role as authoritative guides in the fight against the pandemic, and HWs became a trustworthy and dependent media image in the minds of the people, creating a good doctor-patient atmosphere, which, together with positive media coverage, somewhat eased the previously tense doctor-patient relationship (37). Therefore, the doctor-patient relationship was much improved during the pandemic, which could reduce the risk of clinician-patient conflicts, and protect HWs from PVV.

### Recommendations for the protection of HWs’ occupational health

The following recommendations based on this study should be considered. First, during the pandemic, the workplace risk assessment should be updated regularly for each specific setting, as well as for each role, task, or set of tasks to determine the level of risk for potential occupational exposure related to different jobs, work tasks, and work settings, and to plan and implement adequate IPC measures for risk prevention and mitigation (1). Second, during outbreaks of epidemics, measures to strengthen the occupational protection of HWs should be taken, especially to ensure a reasonable workload for HWs, reasonably arrange shifts and compensatory leave for medical staff, establish a long-term mechanism to protect medical staff, and create a good atmosphere of respect for medical care (38). Third, when adopting IPC measures, hospitals should reduce aggregation, relieve HWs’ work pressure, and carry out patient-centered medical services by improving the consultation system (39), promoting online treatment on the Internet, and reasonably coordinating outpatient clinic resources.

## Limitation and strength

First, the PVV data set was drawn from the Notification system and is surveillance data, recording the more severe PVV, which is somewhat different from the PVV obtained from general cross-sectional studies. Second, while the DID model requires data for at least 1 year before and after the implementation of the measures, the data for this study were collected in early 2021 and the outbreak occurred in late 2019, which corresponds to a year of change that had already occurred. Third, we have only tracked the data for 1 year after the implementation of the policy, which is only short-term data and belong to the immediate impact, not the long-term effect. We will collect longer data to observe the trend of PVV in the future. Nevertheless, this study has five consecutive years of data that can be used for DID modeling and validate the question of common trends prior to measure implementation, as well as conduct placebo tests to exclude the effects of other policies, which is relatively rarely done in PVV studies.

## Conclusion

During the period of COVID-19, a series of measures were formulated and implemented in China to prevent and control infection and transmission, as well as protect the occupational health and safety of HWs. The multi-dimensional and comprehensive IPC measures throughout the pandemic in China have not only controlled the pandemic, but also directly or indirectly reduced the incidence rate of PVV by alleviating the stress of HWs and the crowded working environment, creating a good order of admission, and reducing patient waiting time.

## Data availability statement

The raw data supporting the conclusions of this article will be made available by the authors, without undue reservation.

## Ethics statement

This study was approved by Ethical Review Committee of Chinese Academy of Medical Sciences (IMICAMS/8/22/HREC). PHIs directors were informed personally about the study by the principal investigator and were supportive of the study. Written informed consent was obtained from all participants before the study. To ensure anonymity, no names or other identifiers were used.

## Author contributions

JH conceived and designed the study and modified the manuscript. KS reviewed the literature, collected the data, performed the data analysis, modified the manuscript, provided important insights in response to the discussion, and drafted the final manuscript. CZ conducted the literature search, made significant contributions to the literature review and background, assisted with data analysis, interpreted the data results, and wrote the first draft. All authors contributed to the article and approved the submitted version.

## Funding

This research was funded by the National Natural Science Foundation of China (Project Identification Code: 71804192).

## Conflict of interest

The authors declare that the research was conducted in the absence of any commercial or financial relationships that could be construed as a potential conflict of interest.

## Publisher’s note

All claims expressed in this article are solely those of the authors and do not necessarily represent those of their affiliated organizations, or those of the publisher, the editors and the reviewers. Any product that may be evaluated in this article, or claim that may be made by its manufacturer, is not guaranteed or endorsed by the publisher.
